# Inner Plant Values: Diversity, Colonization and Benefits from Endophytic Bacteria

**DOI:** 10.3389/fmicb.2017.02552

**Published:** 2017-12-19

**Authors:** Hongwei Liu, Lilia C. Carvalhais, Mark Crawford, Eugenie Singh, Paul G. Dennis, Corné M. J. Pieterse, Peer M. Schenk

**Affiliations:** ^1^School of Agriculture and Food Sciences, The University of Queensland, Brisbane, QLD, Australia; ^2^Hawkesbury Institute for the Environment, Western Sydney University, Penrith, NSW, Australia; ^3^Centre for Horticultural Science, Queensland Alliance for Agriculture and Food Innovation, The University of Queensland, Brisbane, QLD, Australia; ^4^Department of Natural Resources and Mines, Toowoomba, QLD, Australia; ^5^School of Earth and Environmental Sciences, The University of Queensland, Brisbane, QLD, Australia; ^6^Plant-Microbe Interactions, Institute of Environmental Biology, Department of Biology, Faculty of Science, Utrecht University, Utrecht, Netherlands

**Keywords:** biocontrol bacteria, endophytic bacteria, plant defense signaling, plant growth promotion, plant microbiome

## Abstract

One of the most exciting scientific advances in recent decades has been the realization that the diverse and immensely active microbial communities are not only ‘passengers’ with plants, but instead play an important role in plant growth, development and resistance to biotic and abiotic stresses. A picture is emerging where plant roots act as ‘gatekeepers’ to screen soil bacteria from the rhizosphere and rhizoplane. This typically results in root endophytic microbiome dominated by Proteobacteria, Actinobacteria and to a lesser extent Bacteroidetes and Firmicutes, but Acidobacteria and Gemmatimonadetes being almost depleted. A synthesis of available data suggest that motility, plant cell-wall degradation ability and reactive oxygen species scavenging seem to be crucial traits for successful endophytic colonization and establishment of bacteria. Recent studies provide solid evidence that these bacteria serve host functions such as improving of plant nutrients through acquisition of nutrients from soil and nitrogen fixation in leaves. Additionally, some endophytes can engage ‘priming’ plants which elicit a faster and stronger plant defense once pathogens attack. Due to these plant growth-promoting effects, endophytic bacteria are being widely explored for their use in the improvement of crop performance. Updating the insights into the mechanism of endophytic bacterial colonization and interactions with plants is an important step in potentially manipulating endophytic bacteria/microbiome for viable strategies to improve agricultural production.

## Introduction

It has been projected that the world’s population will increase to 9.1 billion by 2050 (FAO, 2009). Increasing agricultural productivity is of the upmost priority for governments around the globe. However, the pathway to achieving this goal is becoming progressively difficult. Reduced arable land through urban sprawl, climate change and poor land management practices has led researchers and practitioners to explore non-traditional farming practices (Smith et al., 2016). The purposeful use of plant growth promoting bacteria (PGPB) as biofertilizers in agriculture is a promising technology to provide effective and environmentally friendly solutions with the potential to ensure food security (Glick, 2014). However, to achieve this, scientists still need to forge a deeper understanding of the mechanisms underlying plant growth benefits by PGPB (e.g., beneficial endophytes).

Millions of years of evolution have led plants to develop a diverse range of mechanisms to cope with biotic and abiotic stresses. Establishing a continuing relationship with bacteria has evidently enhanced their capability to cope with stresses as well as to facilitate their growth and development. Endophytes are non-pathogenic organisms that live inside plant tissues for at least part of their life cycles (Rosenblueth and Martínez-Romero, 2006). Some endophytic bacteria are able to systemically prime the plant’s immune system. Primed plants do not display major changes in defense-related gene expression in the absence of a pathogen, but mount an accelerated defense response upon pathogen or insect attack, providing broad-spectrum resistance (Pieterse et al., 2014; Conrath et al., 2015). Recently, several studies demonstrated the effectiveness of endophytic bacteria in protecting plants from a series of abiotic stresses including drought (Rolli et al., 2015; Sheibani-Tezerji et al., 2015), low temperature (Su et al., 2015; Subramanian et al., 2015) and salinity (Ali et al., 2014). In addition, it was also observed that *Agave tequilana* plants directly digested endophytic bacteria for a nitrogen (N) source for their growth (Beltran-Garcia et al., 2014). Therefore, a potential to explore the development of endophytic bacteria in agricultural practices and in variable climatic conditions should not be ignored.

The interaction of the drivers of plant root microbial habitat and diversity have been explored in comprehensive reviews such as; Bulgarelli et al. (2013), Gaiero et al. (2013), and Reinhold-Hurek et al. (2015). The influence of climate change on these microbial communities and how they react and adapt to these changes as well as the use of endophytic bacteria in agriculture have been identified as potential research priorities. In order to fully understand these influences, the mechanisms behind the techniques used and potential drivers of inovation need to be explored. An underlining goal of this review is to give a brief understanding of the biodiversity, distribution and elements of endophytic bacteria to lay a platform for exploring their potential benefical use in agricultural practices. Exploring the plant growth promoting traits of endophytic bacteria, such as boosting plant nutrient uptake or buffering capacity from abiotic stress is a relatively novel but promising area for the development of sustainable agriculture (Glick, 2014; Santoyo et al., 2016).

In this review, the authors aim to discuss key issues within the scope of plants and endophytic bacteria interactions. Based on the findings in most recent studies on endophytic bacteria, we explore (1) which bacteria live in plant endophytic habitats, (2) how do endosphere bacterial communities respond to plant stresses and environmental stimuli, (3) where exactly do endophytic bacteria colonize plants, (4) what are the traits that enable bacteria to successfully invade and persist into standing heterogeneous communities, (5) how do bacterial endophytes deal with the plant’s immune system, (6) how does the plant host influence endophytic colonization via hormone signaling pathways, and (7) what are the traits of endophytic bacteria that deliver plant phenotypes and therefore may hold promise for use in agriculture. We believe that understanding the interactions between endophytic bacteria and their plant hosts will assist in the design of new strategies for productive and sustainable practices in agriculture.

## Biodiversity and Acquisition of Endophytic Bacteria

Different plant organs are associated with different endophytic bacterial communities in terms of diversity and composition. The microbiome in the root endosphere is significantly less diverse than microbiomes in the rhizosphere and bulk soil (Liu et al., 2017). The number of bacterial cells within root endophytic environments reaches c. 10^4^–10^8^ per gram of root tissues, which is considerable less when compared with bulk soil (c. 10^6^–10^9^ bacterial cells g^-1^ soil) and rhizosphere (c. 10^6^–10^9^ bacterial cells g^-1^ plant tissue) (Bulgarelli et al., 2013). This suggests that roots are effective habitat filters, restricting community membership to progressively more narrowly defined lineages as environments deviate from soil to roots (Bulgarelli et al., 2012). Root endophytic bacterial communities are typically dominated by Proteobacteria (∼50% in relative abundance), Actinobacteria (∼10%), Firmicutes (∼10%) and Bacteroidetes (∼10%) (Supplementary Table S1). Other bacterial phyla, including but not limited to Chloroflexi, Cyanobacteria, Armatimonadetes, Verrucomicrobia, Planctomycetes, and Nitrospirae are common in the root endosphere, but represent a smaller fraction of the community (Supplementary Table S1) (Sessitsch et al., 2012; Edwards et al., 2015). Archaea, Acidobacteria and Gemmatimonadetes appear to be either absent or rare (<1%) in the root endosphere despite being significant representatives of bulk soil microbial communities (Bates et al., 2011; Sessitsch et al., 2012).

Understanding the differences, if any, between plant root and leaf/shoot endosphere microbiomes is the first step of many in developing a clear pathway to beneficial development of biofertilizers in agriculture. There is evidence that plant root bacterial endophytes are mainly recruited from soil, which then ascend to stems and leaves via the apoplast in xylem vessels (Chi et al., 2005). Therefore, it is not surprising that plant leaf/shoot endosphere microbiomes have significant overlaps with those in roots at both, the taxonomic and functional levels (Bodenhausen et al., 2013; Bulgari et al., 2014; Bai et al., 2015). Consistently, the work of de Oliveira Costa et al. (2012) showed that Proteobacteria, Actinobacteria and Firmicutes are the dominant groups in the leaf microbiome of common bean plants (*Phaseolus vulgaris*), which was revealed by a culture-based analysis.

Recent studies observed that the plant root endosphere could be dominated by only a few bacterial groups, which provides further evidence of the active and robust selection of bacteria from soil to plants. Examples for this are Gammaproteobacteria of the genera *Enterobacter, Pseudomonas* and *Stenotrophomonas* that constitute the core bacterial operational taxonomic units (OTUs) in root endosphere of both, the sweet potato IPB-137 (Gammaproteobacteria, 47%) (Marques et al., 2015) and rice (Gammaproteobacteria, 30–98%) (Sessitsch et al., 2012; Ferrando and Scavino, 2015; Ren et al., 2015a). In some cases, it was found that only one or two bacterial OTUs dominated the endosphere of plant tissues. These are a *Pseudomonas*-like OTU (34%) in the roots of *Populus deltoids* (Gottel et al., 2011) and two OTUs affiliated to *Pseudomonas* (52%) and *Enterobacter* (35.5%) in sugarcane stems (Magnani et al., 2013). Members of Actinobacteria, especially the genus *Streptomycetes* are well known for their efficient synthesis of antibiotic compounds that suppress a diverse range of phytopathogens (Palaniyandi et al., 2013). Metagenomic surveys using 16S rRNA phylogenetic marker gene showed that the *Streptomycetaceae* family typically dominated the Actinobacteria in the root endosphere of both *Arabidopsis* (Bulgarelli et al., 2012; Lundberg et al., 2012) and wheat seedlings (Liu et al., 2017). Collectively, the abovementioned studies demonstrate that the microbiome in the plant endosphere is much simpler than that in the adjacent soil, and that it harbors distinct assemblages rather than random subsets of the soil microbiome.

This leads to the question of how plants manage to recruit ‘good bugs’ that they might use while expelling those that do not provide benefits. In recent reviews by Bulgarelli et al. (2013) and Reinhold-Hurek et al. (2015), two- and three-step models were proposed for describing the dynamics of the root-associated microbiome across the three niches (rhizosphere, rhizoplane, and endosphere), which highlighted a screening role of each of the root compartments in the acquisition process of the endosphere microbiome. The rhizosphere as the ‘growth chamber’ is the first compartment that profoundly influences the soil microbiome. The distinct physicochemical and biological conditions caused by the carbon-rich molecules and antimicrobial compounds in the rhizosphere may favor the growth and reproduction of some soil bacterial groups while suppressing some others. Secondly, the root rhizoplane plays a ‘critical gating role’ (Edwards et al., 2015). Those bacteria that are attracted to the rhizosphere but lack adhesion ability are not permitted to enter the endosphere due to their inability to bind to root surfaces (Reinhold-Hurek et al., 2015). Therefore, only a small subset of the rhizosphere microbiome enters the endosphere. Lastly, the plant immune system actively excludes some specific bacterial groups (Lundberg et al., 2012). As observed in many cases, Acidobacteria, Gemmatimonadetes and Archaea consecutively decrease while Proteobacteria (especially the Gammaproteobacteria) significantly increase in relative abundance from bulk soil to root endosphere (Edwards et al., 2015; Liu et al., 2017). The underlying mechanisms and the ecological rationale behind this phenomenon are still poorly understood. Despite being still speculative, distinct pH and nutrient conditions (Naether et al., 2012) as well as the high [O_2_] (Blossfeld et al., 2011) in the root interior may be major factors leading to the rare presence of Acidobacteria in plant roots. It is worth pointing out, nevertheless, that the bacterial microbiome in the plant endosphere is not likely to be simply assembled by the plant, but is also the result from complex microbial interactions (e.g., microbial competition and cooperation). Overall, more research into how the endosphere microbiota are assembled is necessary to shed light onto strategies for recruiting, maintaining and monitoring them for the provision of benefits to sustainable agriculture.

## Determinants of Microbial Community Assembly in the Endosphere

The bacterial components in plant interior are mostly harmless or beneficial to their host and they are dynamic (Rosenblueth and Martínez-Romero, 2006). The changes of their composition and diversity are driven by the ecology of the plant and soil, which are highly dependent on the plant’s geographic location, endogenous host interactions and exogenous environmental factors (Edwards et al., 2015). Soil that harbors an immensely rich pool of bacterial species is the microbial ‘seed bank’ for roots, and its properties may affect plant physiology and root exudation profiles which in turn profoundly influence the structure of the root endosphere microbiome (Philippot et al., 2013). Studies performed on the endosphere microbiome of different plants, using high-throughput amplicon sequencing, have revealed that host plant species (Shen and Fulthorpe, 2015; Ding and Melcher, 2016), genotype (Marques et al., 2015; Rodriguez-Blanco et al., 2015), plant organ type (Hameed et al., 2015), developmental stage (e.g., seedling or mature plant) (Ren et al., 2015a; Yu et al., 2015; de Almeida Lopes et al., 2016), growing season (e.g., of trees) (Shen and Fulthorpe, 2015; Ding and Melcher, 2016), geographical location (field conditions) (Edwards et al., 2015), soil type (Edwards et al., 2015), host plant nutrient status (Hameed et al., 2015), cultivation practice (Edwards et al., 2015) and fertilization (Rodriguez-Blanco et al., 2015) are among the observed factors that significantly influence the plant endosphere microbiome.

It was observed that transgenic glyphosate-resistant cultivars of soybean had a higher diversity and abundance of culturable endophytic bacteria than wild-type plants (de Almeida Lopes et al., 2016). Marques et al. (2015) depicted that the plant genotype affected the functional diversity of endophytic bacteria, as IAA-producing strains were predominantly isolated from one of the three genotypes of sweet potato studied. The work of Kõiv et al. (2015) also demonstrated that plant diseases can influence the composition of endophytic bacterial communities. An anaerobic pectolytic *Clostridia* population was particularly enriched in soft rot disease- (caused by *Pectobacterium atrosepticum*) infected potato (*Solanum tuberosum*) tubers, and this change occurred possibly due to oxygen depletion inside the tubers (Kõiv et al., 2015).

In addition to soil and host properties, fluctuations of environmental CO_2_ and temperature modulate endophytic bacterial communities. In the context of climate change and given the importance of endophytic bacteria for plant growth and health, understanding how endophytic bacteria respond to an elevated CO_2_ or temperature can aid in future decision-making policies around environmental issues. Ren et al. (2015b) demonstrated that leaf endophytic bacteria appear to be more vulnerable to climate change than soil bacterial communities. The community structure of endophytic bacteria in rice leaves was influenced by elevated CO_2_ levels at the tillering and filling stages, but not during maturity, and this influence also correlated with N fertilization levels (Ren et al., 2015a). Moreover, Ren et al. (2015b) showed that endophytic communities inhabiting leaves at different locations (upper or lower leaf) in the plant respond differentially to elevated CO_2_. Oxygen availability also exerts effects on endophytic bacterial communities in rice, especially on diazotrophs. Ferrando and Scavino (2015) observed that diazotrophic community composition in rice roots shifted significantly after flooding stress, with Gammaproteobacteria and Betaproteobacteria being the predominant groups in the endosphere before and after flooding. These results are intriguing as they indicate a restructuring of microbe populations in the endosphere by plants upon changes in specific environmental factors. However, the challenge remains to discover whether there is a link between corresponding microbiome variation and plant physiological conditions/health.

In order to address the question of ‘which bacteria live in the endosphere?’, taxonomy-based approaches were deployed. By contrast, function-based metagenomics, metatranscriptomic, and metaproteomic analyses which represent the functional variations of endophytic communities are able to answer ‘what can they do in the endosphere?’ Current investigations on the functional dynamics of endophytic communities using metagenomic analyses have been performed to a much lesser extent than phylogeny-based analyses. Recently, a functional study conducted on tomato plants revealed that bacterial endophytes colonizing roots were significantly affected by root-knot nematodes (Tian et al., 2015). Genes involved in plant polysaccharide degradation, carbohydrate/protein metabolism and N_2_ fixation were increased in abundance, which indicates that bacteria inside roots may start proliferative growth and become saprophytic after infection by root-knot nematodes. This study also provides evidence to suggest that particular functional attributes of endophytic bacteria are induced in plants suffering from stress.

## Distribution of Endophytic Bacteria and Colonization Patterns

**FIGURE 1:**
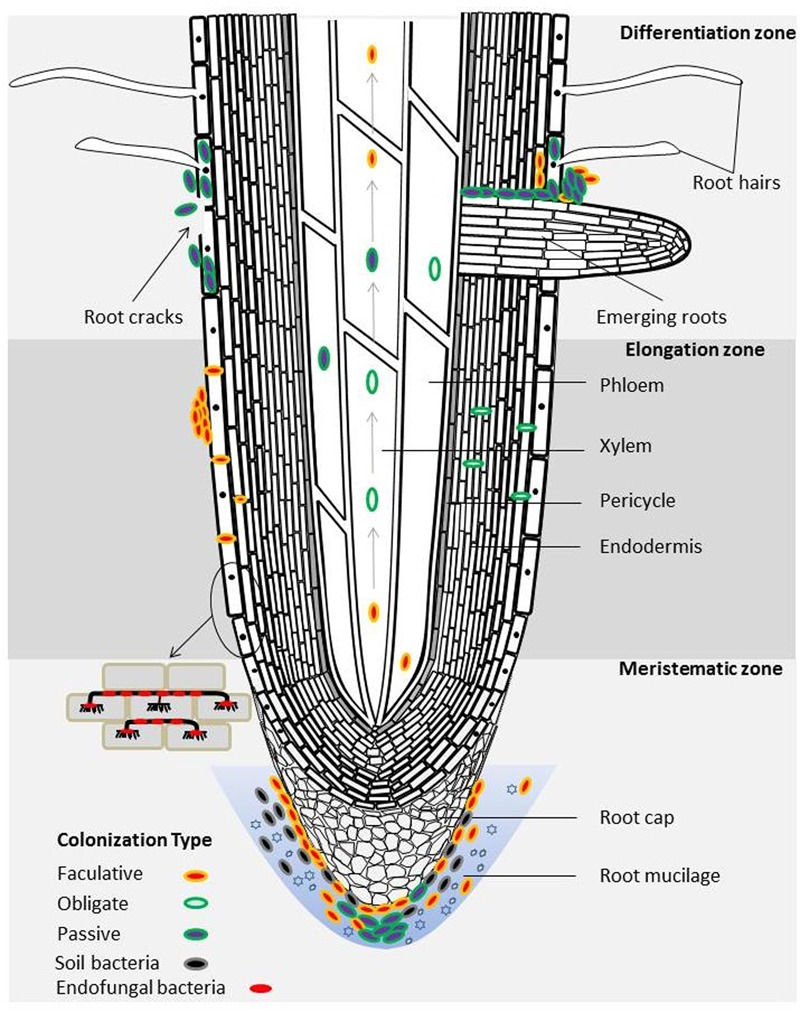
Schematic representation of the bacterial distribution and colonization patterns in the endosphere of a plant root. The emerging sites of lateral roots are among the hotspots of bacterial colonization. Arrows represent the translocation of bacteria inside the xylem and phloem. Endophytic bacteria may engage in different life styles as depicted by different colored ovals. This illustration was inspired by studies of Compant et al. (2005, 2008) and Glaeser et al. (2016).

Bacterial colonization patterns in plant endophytic compartments have thus far been mainly studied in grasses (e.g., rice and kallar grass) using cultivated model strains. Some of the most popular approaches to enumerate and visualize colonization of bacteria in plant tissues include fluorescence *in-situ* hybridization (FISH) and using reporter gene- (e.g., *gfp* or *gus*) modified bacterial strains combined with microscopy. In plants, emerging lateral roots break through the epidermis, cortex, endodermis, casparian strip (band around endodermis) and pericycle, thereby naturally forming a ‘highway’ for bacteria to enter at these sites. From there, bacteria can further enter the phloem and xylem vessels that transport photosynthates (phloem), nutrients and water (xylem) (a schematic illustration is shown in **Figure 1**) (Compant et al., 2010). Bacteria colonizing inside the root conductive tissues can further translocate to shoots and leaves driven by plant transpiration (Compant et al., 2010). Endophytic infection can also occur at wounds (e.g., leaf scars, root ruptures) as a result of herbivore or other mechanical damage (Compant et al., 2010).

Typical hot spots for bacterial colonization are lateral root emergence sites, outer cell layers, root cortex, phloem and xylem, which can occur both intracellularly and in the apoplast (Reinhold-Hurek et al., 2006) (**Figure 1**). For instance, PGPB *Burkholderia* sp. strain PsJN colonized root rhizodermis cells, internal tissues, particular internodes and leaves of grapevine (Compant et al., 2005, 2008). The work of Anand and Chanway (2013) and Anand et al. (2013) also supports this in their findings for the diazotrophic bacterial strain *Paenibacillus polymyxa* P2b-2R which extensively colonized the surface and interior of roots, stems and needles of lodgepole pine (*Pinus contorta* Dougl. var. *latifolia* Engelm.). An unusual colonization strategy has been recently discovered for a facultative intracellular symbiont of *Methylobacterium extorquens* strain DSM13060, which aggregated around the nucleus of the living cells of Scots pine (*Pinus sylvestris* L.) shoot tips (Koskimäki et al., 2015). Broadly speaking, the plant parts in or close to soil are inclined to harbor more bacteria than the uppermost plant organs (Fisher et al., 1992).

**FIGURE 2:**
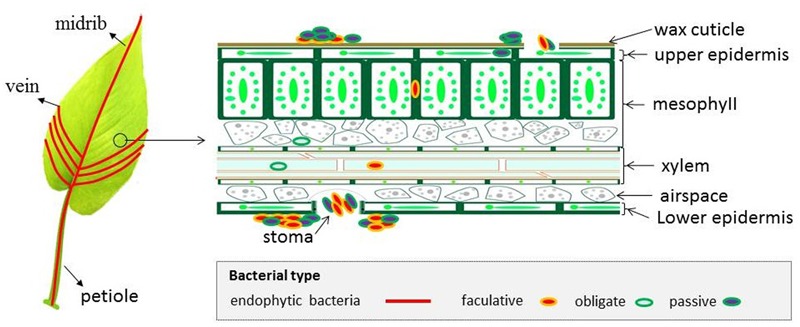
Schematic representation of bacterial colonization patterns in a leaf. The picture shown on the left demonstrates that the presence of bacteria has been detected in the leaf petiole, midrib and veins. The picture shown on the right is a magnified leaf cross-section, which demonstrates that endophytic bacteria may not only colonize the apoplast but are also present intracellularly. Endophytic bacteria are believed to be able to ascend from roots to leaf via the vascular tissues of xylem and phloem.

Regarding bacteria in the phylosphere, there are indications that bacterial endophytes are derived from soil by screening soil bacteria via rhizosphere and root systems (Lamb et al., 1996). Alternatively, these bacteria can be from phyllosphere epiphytes through natural openings (e.g., stomata, hydathodes), wounds and cracks generated by wind, insect and pathogen attacks (Vorholt, 2012). Specifically, in a leaf, bacteria can colonize in upper epidermis cells, palisade mesophyll cells, xylem vessels as well as spaces between spongy mesophyll layer cells (Olivares et al., 1997) (**Figure 2**). Bacteria have also been detected in plant reproductive organs, such as flowers, fruits and seeds, but in small numbers (Rosenblueth and Martínez-Romero, 2006; Compant et al., 2011; Truyens et al., 2015). Consistent with this, Compant et al. (2011) visualized the colonization of *Pseudomonas* spp. and *Bacillus* spp. in grapevine using FISH and found these bacteria colonizing epidermis and xylem of the ovary, intercellular spaces of pulp cells and along cell walls inside seeds. Another example is *Streptomyces mutabilis* strain IA1 that controls the fungal pathogen *Fusarium culmorum* in wheat seedlings and colonizes the area inside the caryopsis, up to the endocarp layer of wheat seeds (Toumatia et al., 2016).

Colonization of endophytic bacteria can be also categorized into ‘obligate,’ ‘facultative’ and ‘passive’ depending on if they require plant tissue to live and reproduce (for a review on this topic see Hardoim et al., 2008). Obligate endophytic bacteria are derived from seeds and cannot survive in soils. Facultative endophytic bacteria widely exist in soil, and they carry out colonization and infection when conditions are suitable. Most facultative endophytic bacteria remain within the cortex but some also enter the central phloem and xylem (Compant et al., 2010). Bacteria lacking the capability to colonize and infect can enter plant endophytic niches via wounds and cracks on the plant, which is documented as the passive mode of endophytic colonization (Christina et al., 2013) (**Figure 1**). Current evidence reveals that some bacteria live in symbiosis with plant endophytic fungi (Desirò et al., 2015; Glaeser et al., 2016). Interestingly, particular endofungal bacteria colonize plants in a similar fashion as their fungal host (**Figure 1**). For instance, the endofungal bacterium *Rhizobium radiobacter* F4 hosted by the fungus *Piriformospora indica* colonizes plant roots and forms aggregates of attached cells and dense biofilms at the root surface (Glaeser et al., 2016).

In summary, it is evident that bacteria are able to colonize both intracellularly and extracellularly the interior of plants. Despite having been detected in all plant parts, roots that have the most intimate contact with soil may function as the first avenue for the recruitment of endophytic bacteria. Endophytic bacteria may have a genetic basis to their different colonization and infection patterns, which may further correlate to their interaction patterns within plants. In the following section, we provide details on traits that enable endophytic bacteria to successfully establish in their plant hosts.

## Traits for Successful Invasion, Colonization and Translocation of Endophytes

During millions of years of coevolution with plants, bacteria have been equipped with necessary traits that enable them to invade, colonize and translocate in the plant’s interior. Motility, chemotaxis, production of cell-wall degrading enzymes and lipopolysaccharide formation are among the observed traits for bacteria to infect and adapt to life inside plants (Piromyou et al., 2015). The importance of these traits has been confirmed by comparative genomics, metagenomics and transcriptomic analyses, combined with mutational studies (Böhm et al., 2007; Straub et al., 2013; Sheibani-Tezerji et al., 2015). Bacteria may adjust gene expression when infecting and colonizing plants (Coutinho et al., 2015; Piromyou et al., 2015). This can be demonstrated by genes encoding proteins related to bacterial motility, chemotaxis and adhesion that were induced in *Burkholderia kururiensis* M130 in the presence of rice plant extracts (Coutinho et al., 2015). The bacterial flagellum that often acts as a potent microbe-associated molecular pattern (MAMP) for recognition by the innate immune system may also mediate endophytic competence by enabling bacterial chemotactic movement and anchoring to plant surfaces (Buschart et al., 2012). The five endophytic bacteria examined by Straub et al. (2013) all contain the entire flagella machinery and a flagella-deficient mutant was hampered in colonization efficiency of wheat roots (Croes et al., 1993). Additionally, adherence to the root surface is also a crucial step for bacteria to infect plants. This is exemplified by the fact that genes encoding Type IV pili (TFP), the crucial virulence factor formed by pilin subunits, exist in the genome of endophytic *B. phytofirmans* PsJN bacteria (Mitter et al., 2013). Moreover, mutant analysis has demonstrated the essential role of TFP-dependent adhesion for the establishment of *Azoarcus* sp. inside rice roots (Dörr et al., 1998). It was further revealed that TFP retraction protein-mediated twitching motility is essential for N_2_*-*fixing bacteria *Azoarus* sp. strain BH72 to establish inside rice roots but this was not important for the colonization on the root surface (Böhm et al., 2007).

Cell-wall degrading enzymes are important for plants to break plant cell walls and translocate compounds to the apoplast. Genes encoding cell-wall degrading enzymes widely exist in the genomes of endophytic bacteria (Straub et al., 2013). For example, genes encoding plant polymer-degrading cellulases, xylanases, cellobiohydrolases, endoglucanase and cellulose-binding proteins were detected in high copy numbers in the metagenome of rice root endophytic bacterial communities (Sessitsch et al., 2012). *In vitro* assays confirmed that endoglucanases are crucial for *Azoarcus* sp. to colonize inside rice roots (Reinhold-Hurek et al., 2006). To be able to ingress intracellularly and translocate within the symplast, endophytic bacteria may also secrete pectinases to degrade the middle lamella between plant cells. It was found that pectinase is an important determinant modulating early infection of the PGPB *Bradyrhizobium* sp. SUTN9-2 on rice, which originally formed symbiotic relationships with the leguminous weed *Aeschynomene americana* (Piromyou et al., 2015). Moreover, pectin esterase expression in this bacterium was up-regulated after being inoculated on rice seedlings (Piromyou et al., 2015). In addition to the above-mentioned traits, Kost et al. (2014) found that oxalotrophy, the capacity of utilizing oxalate as a carbon source, is required for the successful colonization of *B. phytofirmans* PsJN on lupin and maize plants. Oxalotrophy was reported to be only associated with plant-beneficial *B. phytofirmans* species, while plant pathogenic or human opportunistic pathogenic species of the *Burkholderia* genus are not able to use oxalate (Kost et al., 2014). This study suggests a role of oxalate in plant selection for beneficial endophytes, while avoiding pathogenic bacteria from the complex soil bacterial communities. Overall, the traits discussed above seem to be required for the active invasion and systemic transmission of endophytic bacteria within plants.

## Bacterial Endophytes Circumvent Host Defense

Plants highly rely on their sophisticated defense systems to counteract attacks from phytopathogens (Jones and Dangl, 2006). MAMP-triggered immunity (also known as horizontal resistance) that has pattern-recognition receptors as a surveillance system to perceive conserved MAMPs equips plants with a first line of basal defense that is able to halt the growth of most pathogens. During the coevolution with plants, pathogens developed the strategy of injecting effectors into plants to suppress or circumvent MAMP-triggered immunity. In response, plants developed a second line of defense called effector-triggered immunity (also known as vertical resistance). Within the latter strategy, plants have developed receptors that recognize the effectors of pathogens. In the case of biotrophic or hemibiotrophic pathogens that depend on living cells for nutrient uptake, a hypersensitive response (HR) may be activated leading to programmed cell death (PCD) of cells under attack (Jones and Dangl, 2006). However, this rapid defense response must be suppressed in the case of necrotrophic pathogens (nutrient uptake from dead or degraded plant tissues) or beneficial microbes, including beneficial endophytes. In many plants, including *Arabidopsis*, the salicylic acid (SA) defense signaling pathway targets biotrophic pathogens, while the jasmonic acid (JA) pathway suppresses the SA pathway playing a role in defense against necrotrophic pathogens, but also insects and beneficial plant-microbe interactions (Pieterse et al., 2012).

The plant immune system can therefore play a major role to influence the colonization and multiplication of plant bacterial endophytes. There is growing evidence that, to avoid antagonistic effects, endophytic bacteria produce their own MAMPs (unlike phytopathogens), which generally do not elicit significant plant immune responses, such as the expressions of pathogenesis-related (PR) proteins (Vandenkoornhuyse et al., 2015). This therefore avoids bacteria being eliminated by the plant’s immune system. Many cell surface components of endophytic bacteria are distinct from those of phytopathogens. For example, the flagellin-sensing system flg22-Flagellin Sensing 2 (FLS2) in grapevine differentially recognizes the flagellin-derived epitopes of endophytic PGPB *B. phytofirmans* from those of a bacterial pathogen such as *Pseudomonas aeruginosa* or *Xanthomonas campestris* (Trdá et al., 2014). This suggests that the flagellin of endophytic bacteria may have evolved to circumvent recognition by the plant’s immune system. Bacterial protein secretion systems (SSs) are another group of important cell surface components with a role in host immune modulation. SSs are composed of large protein complexes that transverse the cell envelope and contain a channel mediating the translocation of proteins or protein-DNA complexes (Green and Mecsas, 2016). Eight (Type I SS∼ Type VI SS and Sec, and Tat) and six (Sec, Tat, secA2, Sortase, Injectosome and Type VII SS) different protein SS have been described for Gram-negative and Gram-positive bacteria, respectively (Tseng et al., 2009; Green and Mecsas, 2016).

**FIGURE 3:**
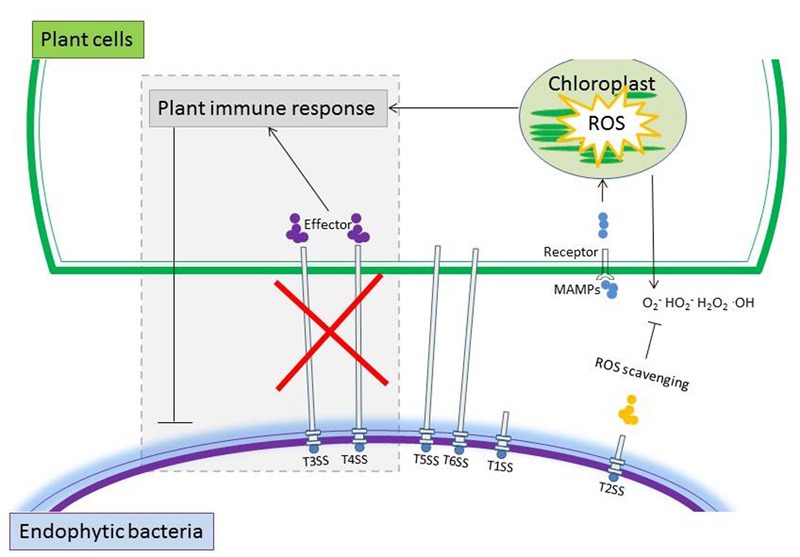
Schematic representation summarizing typical properties that may be employed by endophytic bacteria to cope with the plant’s immune system. Genes encoding secretion systems including T2SS, T5SS, and T6SS are normally detected in high copy numbers in endophytic bacteria. The rare presence of T3SS and T4SS that generally elicit significant plant defense, and the production of scavenging enzymes of endophytic bacteria may have contributed to their successfully colonization in plants.

Among the SSs, T3SS and T4SS are pivotal for pathogens to deliver effector proteins into the plant, which can induce effector-triggered immunity (Green and Mecsas, 2016). T3SS and T4SS may be either absent or present in low abundance in endophytic bacteria and therefore, these bacteria do not seem to elicit significant plant defense responses (**Figure 3**). Krause et al. (2006) sequenced the genome of *Azoarcus* sp. strain BH72 and described it as ‘disarmed’ due to the lack of both T3SS and T4SS as well as other important cell surface components that are usually present in pathogens. Similarly, the genomic inventory of five grass endophytic bacterial strains, including *Herbaspirillum frisingense* GSF30(T), *Gluconacetobacter diazotrophicus* PAI5, *Azoarcus* sp. BH72, *Klebsilla pneumoniae* 342 and *Azospirillum* sp. B510 characterized in biomass grasses completely lack T3SS (Straub et al., 2013). Further, a metagenomic survey demonstrated the rare presence of T3SS- and T4SS-encoding genes in the genomes of eleven endophytic bacterial strains (Reinhold-Hurek and Hurek, 2011). All the endophytic *Herbaspirillum* strains examined so far lack the T4SS that functions in virulence (Juhas et al., 2008; Straub et al., 2013). However, this comes with an exception that T3SS and T4SS are crucial components for *Bradyrhizobium* sp. SUTN9-2 (isolated from the leguminous grass *Aeschynomene americana* L.) to colonize the roots of rice seedlings (Piromyou et al., 2015). With regard to T6SS, their functions are largely unknown but they may also be important for plant-bacterial endophyte interactions (Sessitsch et al., 2012; Mitter et al., 2013). Taken all together, endophytic bacteria tend to lack T3SS and T4SS that in pathogens are related to induction of defense responses but some rhizobium-type endophytic bacteria may require T3SS to establish in the plant endosphere (**Figure 3**).

Production of a range of reactive oxygen species (ROS) is typically a non-specific tactic for plant defense to induce HR and PCD against biotrophic pathogens (Apel and Hirt, 2004). Interestingly, it was observed that colonization of endophytic bacteria also elicited an oxidative burst in rice and the traditional Chinese medicine plant *Atractylodes lancea* (Alquéres et al., 2013; Han et al., 2015; Zhou et al., 2015). To detoxify the initial ROS produced by plants, the endophytic bacteria may resort to ROS-scavenging enzymes (**Figure 3**). A high number and diversity of genes encoding ROS-scavenging enzymes such as superoxide dismutase (SOD) and glutathione reductase (GR) are represented in the metagenome of the endophytic bacterial communities in rice roots (Sessitsch et al., 2012). Genes encoding enzymes involved in ROS-scavenging were also detected in the genome of *Enterobacter* sp. 638 (Taghavi et al., 2010). ROS-scavenging enzymes are reported to be involved in the biological N fixation of *Gluconacetobacter diazotrophicus* and are essential for its successful colonization in endophytic rice roots (Alquéres et al., 2010, 2013). The transcript levels of ROS-scavenging enzyme-encoding genes were upregulated in *G. diazotrophicus* strain PALS when they colonized the plant’s interior (Alquéres et al., 2010, 2013). In summary, endophytic bacteria have evolved a wide range of strategies to avoid, circumvent or cope with the antagonistic effects of plant defenses.

## Plant Hormone Signaling Pathways Influence Endophytic Bacterial Colonization

Given the critical role of phytohormones in plant defense, it is important to determine whether the microbiome is influenced by host plant defense signaling pathways, which is important for at least two reasons. Firstly, these pathways can be induced by external stimuli and have the potential to provide a mechanism to alter the microbiome structure toward plant-beneficial interactions. Secondly, this may help illustrate the role of plant-associated microbiomes in plant nutrition and plant defense against biotic attacks. Several studies have investigated how plant defense signaling regulates the colonization of bacteria inside plants. The activation of the ethylene (ET) signaling pathway suppressed the endophytic colonization of *Medicago truncatula* by the PGPB *Klebsiella pneumoniae* 342 (Kp342) and the human enteric pathogen *Salmonella enterica* serovar Typhimurium (Iniguez et al., 2005). Furthermore, an ET-insensitive *M. truncatula* mutant was ‘hyper-colonized’ by Kp342 compared with wild-type plants (Iniguez et al., 2005). In line with this study, the activation of JA signaling was found to suppress rice root colonization by *Azoarcus* sp. strain BH72 (Miché et al., 2006). The activation of JA signaling also strongly suppressed early stage nodulation in *Lotus japonicus* (Nakagawa and Kawaguchi, 2006). These studies indicate that enhanced plant signaling may restrict the colonization of specific endophytic bacteria or rhizobium in the plant endosphere (**Figure 5**). The suppression of bacterial colonization may be a strategy of the plant’s immune system to control the abundance of hosted bacteria and to maintain the most ‘plant-favorable’ bacterial density in the inner tissues. The potential use of plant hormones for the suppression of specific plant endophytic bacteria warrants further investigation [e.g., to control human pathogens present in food, such as *Salmonella* strains in vegetables (Iniguez et al., 2005)].

**FIGURE 4:**
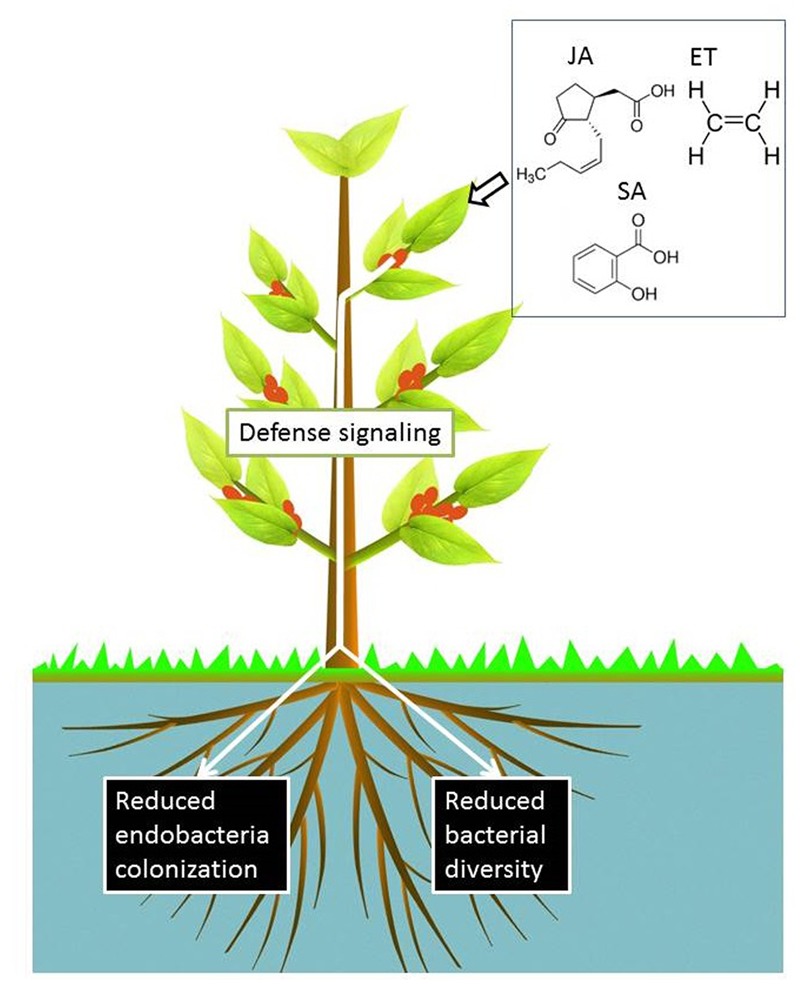
Activation of SA, JA, and ET signaling pathways by exogenous treatments suppresses the colonization of particular bacterial inoculants in the root endosphere. In contrast, defense signaling effects on the plant endosphere-associated microbiome could be small and variable (Iniguez et al., 2005; Miché et al., 2006; Kniskern et al., 2007; Lebeis et al., 2015), but a reduced bacterial diversity has been observed in the roots of wheat seedlings upon activation of JA signaling (Liu et al., 2017).

The diversity of bacterial communities in the endosphere may correlate to plant defense capabilities. This is supported by the higher bacterial diversity in the root endosphere of wilt-resistant tomato cultivar *Arka Abha* than that of the susceptible cultivar *Arka Vikas* (Upreti and Thomas, 2015). Moreover, bacteria isolated from the wilt-resistant cultivar were more likely to employ antimicrobial strategies (e.g., production of siderophores and HCN) than those from the wilt-susceptible cultivar (Upreti and Thomas, 2015). These findings highlight the importance of investigating how the diversity of the endosphere microbiome is affected by plant defense signaling pathways. Our recent study revealed that an activated JA signaling pathway reduced bacterial diversity in the endosphere of wheat roots, while the microbiome in the rhizosphere and shoot endosphere were not influenced (Liu et al., 2017). Similar reports documented that the diversity of endophytic bacterial communities in *Arabidopsis* leaves decreased by the activation of SA signaling, but the communities were not influenced by the activation of the JA-dependent defense pathway (Kniskern et al., 2007). A recent study by Lebeis et al. (2015) provides evidence to suggest that plant roots differentially sculpt their endophytic bacterial communities in different isogenic *Arabidopsis* defense signaling mutants. This observation was based on analysis at the family level and therefore, community profiling at lower taxonomic ranks, that is at genus and species level is required. ET signaling also influences bacterial communities in the plant’s endosphere. It was observed that the diversity of culturable root bacterial communities in isogenic transformed *Nicotiana attenuate* plants impaired in ET biosynthesis (*ir-aco1*) or perception (*35S-etr1*) was lower than that of wild-type plants (Long et al., 2010). Overall, plant signaling defense pathways appear to influence the diversity of endophytic bacteria, although changes could be variable and small (**Figure 4**).

## Plant Growth-Promoting Traits

**FIGURE 5:**
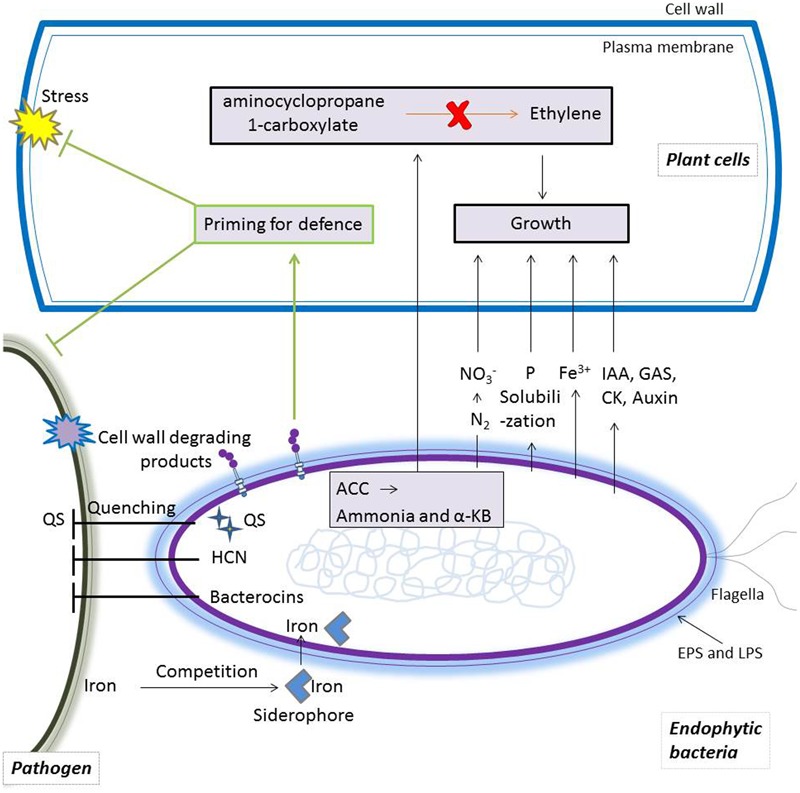
Schematic representation summarizing plant growth promoting traits (PGPTs) of endophytic bacteria. Some endophytic bacteria are able to improve plant growth by reducing the synthesis of stress ethylene in plants, producing growth-promoting phytohormones and providing plants with macro- and/or micro- nutrients such as phosphate, nitrogen and Fe^3+^. Endophytic bacteria can also benefit plants indirectly by suppressing the growth and reproduction of phytopathogens via multi-antagonistic effects, including quenching quorum sensing (QS), competing for nutrients, producing cell-wall degrading products and antimicrobial compounds. In this graph, arrows denote plant-bacteria interactions and ‘⊥’ indicates inhibition. IAA, indoleacetic acid; ACC, 1 aminocyclopropane-1-carboxylic acid; GAs, gibberellins; CK, cytokinin, EPS, extracellular polymeric substance; LPS, lipopolysaccharide; αkb, α-ketobutyrate.

Cropped soils are often deficient in macro and micronutrients and are prone to contain decimating soil-borne pathogens such as *Fusarium, Pythium* and *Phytophthora* ssp (Dixon and Tilston, 2010; Weil et al., 2016). These comprise enormous constrains to plant production worldwide. To obviate this problem and obtain crop yield increase, agricultural production has become increasingly reliant on the use of chemical fertilizers, herbicides, fungicides and insecticides for either supplementing soils with macro and micronutrients or to kill pathogens and insects. However, it is necessary to re-examine many of these approaches due to the potential human and environmental hazards, the intensive energy processes and the depletion of non-renewable resources involved in the industrial production of these agrochemicals (Aktar et al., 2009). Biofertilizers using PGPB is a possible approach to effectively provide plants with nutrients, mediate phytostimulation and therefore reduce the need for chemicals in agriculture, possibly launching a green revolution if better understood and consistent results can be obtained (Lugtenberg and Kamilova, 2009). Endophytic bacteria are capable of promoting plant growth through a wide variety of direct and indirect mechanisms. The direct mechanisms of plant growth promotion include providing plants with nutrients/substrates (e.g., phosphorous, nitrogen and iron) and producing various plant hormones (Santoyo et al., 2016). Indirect beneficial effects of endophytic bacteria on plants are mainly derived from their antagonistic effects toward phytopathogens (Compant et al., 2010). The involved mechanisms for this include production of cell-wall degrading enzymes (e.g., chitinase and β-1,3-glucanase) and antimicrobial compounds, lowering endogenous stress-related ET in plants, induction of induced systemic resistance (ISR) in host plants, quenching the quorum sensing (QS) of phytopathogens and competition for niche and resources (Compant et al., 2010; Glick, 2014; Santoyo et al., 2016). A single endophytic bacterial strain or bacterial community may have more than one of these plant growth-promoting traits (PGPTs) (Rolli et al., 2015; Tsurumaru et al., 2015; Miliute et al., 2016) (**Figure 5**). Bacterial strains with plant growth-promoting functions continue to be discovered but as of yet a clear path to developing PGPTs for agricultural purposes has not been developed (Dey et al., 2014). One reason for the current inconsistency when using bioinocula is that too little is known about the specific interactions that is influenced by the host and microbe genotypes/phenotypes, the environment and whether and how beneficial microbes and microbiomes can be attracted, maintained and adapted to the plant’s requirements. Below, we review the increasingly recognized or novel PGPTs of endophytic bacteria and discuss their potential applications in agriculture.

### Phytohormone Production

Producing phytohormones is a common feature of endophytic bacteria to boost plant growth and increase plant stress tolerance (Pieterse et al., 2009). Genes encoding proteins for biosynthesis of indole acetic acid (IAA) (Zúñiga et al., 2013), cytokinins (CKs) (Bhore et al., 2010) and gibberellins (GAs) (Shahzad et al., 2016) are often present in the metagenome of plant endophytic bacterial communities; e.g., four pathways of IAA biosynthesis were detected in the metagenome of the tomato root gall-associated microbiome (Tian et al., 2015). Inoculation with endophytic bacteria may benefit plants via the production or suppression of phytohormones. For instance, the endophytic bacterium *Sphingomonas* sp. LK11 enhanced tomato growth, which may have been mediated by the production of GAs and IAA (Khan et al., 2014). Additionally, *S. mutabilis* strain IA1 isolated from a Saharan soil was able to produce IAA and GA3. Inoculation of wheat seedlings with this bacterium reduced the progression and severity of *F. culmorum* infection (Toumatia et al., 2016). Another study showed that *Luteibacter* sp. promoted the IAA production by its fungal host, the foliar fungal endophyte *Pestalotiopsis* aff. *neglecta* (Hoffman et al., 2013). This study highlights that there are important indirect plant microbial interactions that promote plant growth that are rarely considered and await discovery. Overall, there is a body of evidence which suggests that enhancing phytohormone production via endophytic bacteria for increased crop production in agriculture is a viable strategy.

### 1-Aminocyclopropane-1-Carboxylate (ACC) Deaminase

The production of ET in stressed plants may lead to decreased plant growth or even cell death when present at high concentrations (Glick, 2014). Some microbes including bacterial endophytes use 1-aminocyclopropane-1-carboxylate (ACC), the immediate precursor of ET, as a carbon and nitrogen source by producing ACC deaminase (Zhang et al., 2011; Karthikeyan et al., 2012; Ali et al., 2014; Glick, 2014). Production of ACC deaminase is arguably the most efficient function for PGPB to reduce the various deleterious environmental effects on plants (Glick, 2014). Increasing global warming, desertification, soil salinization as well as extreme weather events of drought, flood and cold may exert greater stress on plants leading to reduced crop yields (Miraglia et al., 2009). Plants exposed to these stresses accumulate ACC in roots, which systematically spreads to shoots and leaves via the xylem where it is converted to stress ET by ACC-oxidase that is already present in leaves (Tudela and Primo-Millo, 1992). Inoculation with bacterial ACC deaminase producers may decrease the endogenous ACC level in plant roots and therefore increases plant tolerance to stresses (Glick, 2014). A recent study found that bacteria isolated from the endosphere of halophytic *Limonium sinense* (Girard) possessed efficient ACC deaminase activity that were able to increase seed germination, root and shoot length, leaf area and numbers of *L. sinese* seedlings under salinity stress (Qin et al., 2014). While desirable results are often obtained under laboratory conditions, it should be noted that exploration of ACC deaminase producers can only occur in an agricultural context if these bacteria are able to colonize plants persistently. Development of transgenic plants overexpressing ACC deaminase genes also represents a promising strategy to overcome stress ET in plants under stress conditions.

### Cold and Drought Stress Tolerance

The mechanisms underlying endophytic bacteria-mediated improvements of plant resistance to abiotic stress are starting to be elucidated. Tomato plants inoculated with psychrotolerant endophytic bacteria *Pseudomonas vancouverensis* OB155 and *P. frederiksbergensis* OS261 were able to better cope with cold stress (10–12°C) (Subramanian et al., 2015). Less membrane damage and increased antioxidant activity relative to the control plants were observed. Additionally, cold acclimation genes (*LeCBF1* and *LeCBF3*) were induced in bacteria-inoculated plants (Subramanian et al., 2015). Similarly, inoculation of the endophytes *Burkholderia phytofirmans* strain PsJN on *Arabidopsis* led to increased *Arabidopsis* growth and a strengthened cell wall, and thereby an increased cold stress resistance (Su et al., 2015). Endophytic bacteria were also able to increase plant tolerance to drought. Using a transcriptomics approach, it was found that endophytic *B. phytofirmans* PsJN displayed a diverse range of functionalities when inoculated on potato plants (Sheibani-Tezerji et al., 2015). Transcripts involved in transcriptional regulation, cellular homeostasis and ROS detoxification were upregulated in *B. phytofirmans* PsJN in drought stress-affected potato. This suggests that endophytes sense physiological changes in plants and adjust gene expression to adapt to the new environments. Endophytic bacteria have therefore the potential to be used as protective agents in agricultural systems under extreme climatic environments as they can influence plant physiological responses to stresses.

### Boosting Plant Nutrient Uptake

#### Siderophore Production

Although iron is essential for all living organisms, its bioavailability in soil is limited. The production of siderophores by microbes assists plant growth, since these compounds chelate iron in the soil and generate soluble complexes that can be absorbed by plants (Ahmed and Holmström, 2014). We previously found that plants lacking soil bacteria suffered from iron deficiency (Carvalhais et al., 2013). Therefore, this mechanism helps plants to thrive in low iron soils. A great potential for rice root microbiomes in assisting plants in iron uptake has been suggested, given the considerable amount of gene copies encoding proteins in siderophore biosynthesis, siderophore reception and iron storage being detected in the rice root endosphere (Sessitsch et al., 2012). A key role for siderophore production has also been shown for endophytic *Streptomyces* sp. GMKU 3100 (Rungin et al., 2012). Its beneficial properties for rice plants have been established via studying a siderophore-deficient mutant. In addition, siderophores are also involved in plant protection as they deprive phytopathogens of iron by binding to the bioavailable forms of iron first (Verma et al., 2011; Aznar et al., 2015) (**Figure 5**).

#### Nitrogen Metabolism

Nitrogen is crucial for plant growth and health. Approximately 30–50% of the N in crop fields results from biological fixation of N_2_ by soil microorganisms (Gourion et al., 2015). A considerable number of microbial genes involved in N cycling were found in the metagenome of rice roots, which indicates that the rice-related nitrification and ammonia oxidation processes might be subjected to the influence of the endophytic root microbiome (Sessitsch et al., 2012). Some endophytic bacteria possess both, nitrogen fixation (e.g., *nifH*) and denitrification genes (Straub et al., 2013). The importance of endophytic bacteria in N cycling is also supported by the evidence that N_2_ fixation by foliar endophytic bacteria has occurred in many subalpine conifer species (Moyes et al., 2016). For instance, the N fixing isolate *Paenibacillus polymyxa* P2b-2R obtained from lodgepole pine tissue was able to colonize both, the rhizosphere and endosphere compartments, of maize plants and to promote maize growth (Puri et al., 2016). N_2_-fixation by endophytes may provide long-lived conifers with a low-cost and stable way for N supply. However, to which extent do the bacterial endophytes contribute to the whole plant N pool is yet to be investigated.

### Biocontrol of Plant Diseases

Given the similar colonizing patterns as phytopathogens and the intimate contact with plants, bacterial endophytes hold tremendous potential for being used as biocontrol agents in agriculture (Santoyo et al., 2016). For example, biocontrol practices using endophytic bacteria may be achieved either by direct inhibition of pathogens or by indirect strengthening of the plant immune system that in turn halts the growth and development of pathogens in plants (**Figure 5**). Direct inhibition of pathogens is mainly mediated by the synthesis of inhibitory allelochemicals such as antibiotics, hydrogen cyanide (HCN), iron-chelating siderophores and antifungal metabolites (Compant et al., 2010). Quenching QS by degrading autoinducer signals of pathogens is also among the direct modes of biocontrol activity of endophytic bacteria (Miller and Bassler, 2001). Indirect biocontrol mechanisms of endophytic bacteria include the induction of plant systemic resistance that inhibits a broad spectrum of phytopathogens (Niu et al., 2011; Conrath et al., 2015). In this section, we briefly summarized the main biocontrol traits within the abovementioned mechanisms to facilitate the use of endophytic bacteria to combat disease.

#### Primed Plants for Enhanced Defense at Low Physiological Costs

**FIGURE 6:**
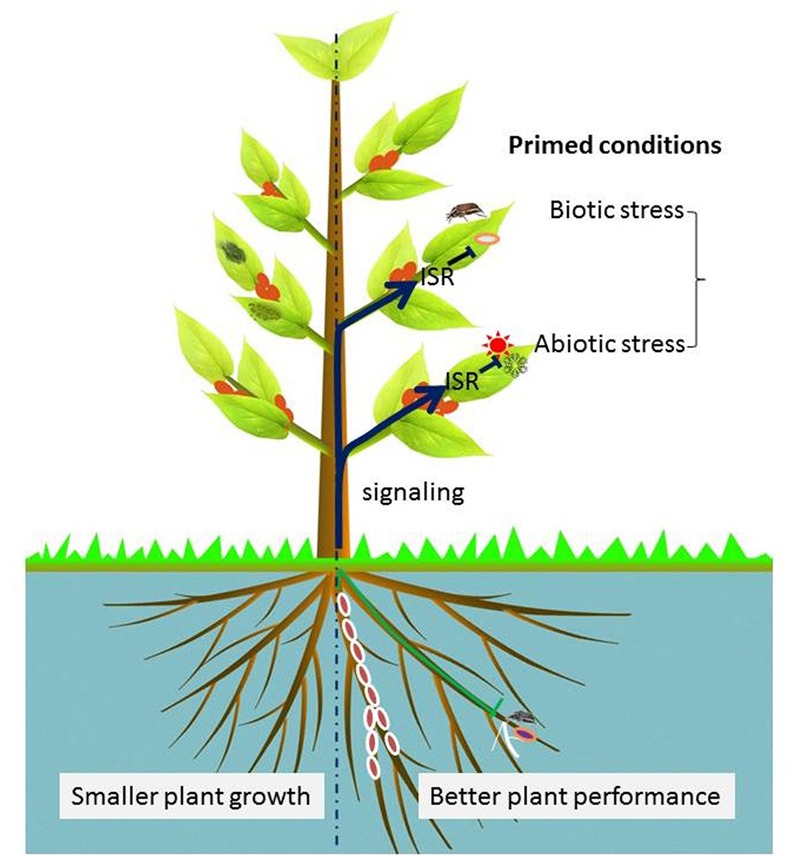
Visualization of endophytic bacteria-induced systemic resistance (ISR). The right half of this illustration presents the elicitation of plant-primed conditions by endophytic bacteria. Endophytic bacteria-mediated ISR may be modulated by either one or combined signaling cascades of SA, JA, and ET in an endophytic bacteria-dependent manner. Some beneficial effects may include changes in root architecture relative to uninoculated plants as shown by the left half of the plant.

Bacterial endophytes have been reported to prime plants for faster and more intense defense responses upon pathogen attacks at low physiological cost to the plant (Martinez-Medina et al., 2016). This process depends on either JA, SA, ET or a combination of these signaling pathways (Pieterse et al., 2014). Typical priming is triggered by exposing plants to a low dose of JA, SA or ET as well as by beneficial plant-microbe interactions (Conrath et al., 2015) (**Figures 5, 6**). For instance, the study by Brock et al. (2013) revealed that *Enterobacter radicincitans* DSM 16656, a highly competitive colonizer of the endophytic environment of various crops, is able to induce priming in *Arabidopsis* via SA- and JA/ET- dependent pathways. Similarly, the endofungal bacterium *R. radiobacter* F4 is able to colonize plant roots without specificity and it is able to increase plant resistance against the bacterial leaf pathogens *Xanthomonas translucens* pv. *translucens* and *Pseudomonas syringae* pv. tomato DC3000. Mutational analysis indicated that the resistance was mediated by ISR via a JA-dependent pathway (Glaeser et al., 2016). All these examples add to a growing number of findings that are paving the way to strategies that use bacterial endophytes to boost plant immunity. However, it remains unclear whether bacteria which colonize the root surface or endosphere contribute to priming and ISR.

#### Antimicrobial Components of Endophytic Bacteria

The antimicrobial compounds produced by endophytic bacteria represent a promising alternative protection to plants against phytopathogens (Brader et al., 2014) (**Figure 5**). For instance, a series of isoforms of iturins were purified from *Bacillus amyloliquefaciens* (Han et al., 2015). Exogenous treatment with the purified iturins induced MAMP-triggered immunity defense in cotton plants; in addition to triggering ROS burst, disrupting cell-wall integrity and affecting fungal signaling pathways (Han et al., 2015). Endophytic bacteria are also able to produce resistance-conferring volatile organic compounds (VOCs) (Chung et al., 2016). Maize plants inoculated with endophytic *Enterobacter aerogenes* that produce VOC 2,3-butanediol (2,3-BD) showed enhanced resistance against the northern corn leaf blight whose causative agent is the fungus *Setosphaeria turcica* (D’Alessandro et al., 2014). The endophytic *Pseudomonas poae* strain RE^∗^1-1-14 that was originally isolated from sugar beet roots was able to suppress the fungal pathogen *Rhizoctonia solani* (Zachow et al., 2015). A novel lipopeptide poaeamide produced by this bacterium may relate to its suppression toward *R. solani* and its establishment in sugar beet roots. Despite the potential scope and impact that these biocontrol traits could have in agriculture, the understanding required for identification of antimicrobial components of bacteria and their application under field conditions is still in its infancy.

#### Interruption of Quorum Sensing of Plant Pathogens

Quorum sensing is a crucial strategy for bacteria to survive in complex ecological niches. It regulates the physiological activities of bacteria, involving cell-to-cell communication, reproduction, biofilm formation, competence and adaptation (Miller and Bassler, 2001). Certain endophytic bacteria employ QS quenching as an antivirulence strategy to control phytopathogens (**Figure 5**). For instance, certain endophytic bacterial strains in *Cannabis sativa* L. disrupt cell-to-cell communication of the biosensor strain *Chromobacterium violaceum* via quenching its QS signals (Kusari et al., 2014). A similar mechanism could be deployed in an agricultural context. For example, diffusible signal factor (DSF) is necessary for the virulence of several *Xanthomonas* species and *Xylella fastidiosa* (Newman et al., 2008). Thereof, *Bacillus* and *Pseudomonas* complemented with *carAB*, a gene required for the fast DSF degradation in *Pseudomonas* spp. strain G, can possibly be used to biocontrol these DSF producing pathogens.

However, a lack of persistence in the context of soil to establish a compatible interaction with plants may mostly make the deployment of endophytic bacteria difficult at field settings (Le Cocq et al., 2017). A much more profound understanding of novel/untapped mechanism of PGPTs in delivering beneficial plant-associated phenotypes is needed to ensure their practicality in the field. Furthermore, genome sequencing of strain collections might foster a faster and less labor-intensive method to screen for sets of PGPTs that are readily detected in genomes of endophytic bacteria.

## Concluding Remarks and Future Prospects

Insights into the microbial ecology of the plant’s endosphere have been greatly expanded in the era of high-throughput DNA sequencing. Phylogenetic marker gene sequencing surveys and meta’omic analyses have enabled scientists to probe microbial community composition in a high-resolution and culture-independent manner. Based on these techniques and culture-dependent methods, solid proofs have been obtained that plants sculpt their root endophytic microbiome, and roots have an effective ‘gate-keeping’ role in this process. Although the inoculation of beneficial bacterial endophytes can notably improve plant growth and yield, it remains unclear if it is essential for these bacteria to colonize internally to generate beneficial effects on plants. Similarly, there are pending questions regarding to which extent endophytic microbiomes support plant growth and defense. For example, how much the nitrogen fixed by endophytic diazotrophs contributes to the overall plant nutrition? Also, whether/how microbial fluctuations in the endosphere correlate to plant health and behavior?

The use of gnotobiotic plants (grown either under sterile conditions or with known microbes) would allow the elucidation of the importance of the endosphere microbiota in plant growth and health. Additionally, it will be of great interest in the future to reveal the mechanisms and ecological rationales behind the rare presence of Acidobacteria, Gemmatimonadetes and Archaea in the plant’s endosphere. These microorganisms are still prohibitively difficult to be cultured. Besides taxonomic surveys, recently emerging techniques like single-cell isolation and sequencing should provide alternatives that circumvent the necessity for cultivation and thereby give steps forward to obtain more comprehensive pictures about their lifestyles and interactions with plants (Gawad et al., 2016). Furthermore, there are some prohibitive technical obstacles for studying the endosphere microbiome. Many laboratories still have difficulties in optimizing DNA samples of surface-sterilized plant tissues (e.g., leave, shoot, seeds) for microbiome sequencing purposes. This is due to largely the abundance of bacterial DNA in non-root tissues being much smaller, relative to plant DNA. Besides optimizing the PCR conditions, a non-biased enrichment of endophytic bacterial cells from plant tissues may circumvent this problem (Dos-Santos et al., 2017).

Although there is a wealth of literature on culture-dependent and independent characterization of endophytic bacterial diversity and the associated *in vitro* mechanisms for plant growth promotion, reports on successful use of endophytic bacteria in plants under field conditions are extremely scarce. Nevertheless, the effect of the *Burkholderia phytofirmans* strain PsJN has been demonstrated to increase biomass and promote growth in switchgrass (*Panicum virgatum* L.) especially in low fertility soils (Lowman et al., 2015). Furthermore, systemic resistance was induced in pepper infected with *Xanthomonas axonopodis* pv. vesicatoria (causal agent of bacterial spot) in the field by an additive effect of the endophyte *Bacillus pumilus* INR7 combined with the chemical inducer benzothiadiazole (Yi et al., 2013).

Proteobacteria, Actinobacteria and Firmicutes are core phyla in the plant endosphere, which are also those groups harboring commensal plant growth-promoting bacteria. Given the diverse PGPTs, the intimate interactions with plants and similar colonizing patterns as phytopathogens, bacterial endophytes bear a great potential to be used for developing biocontrol agents and biofertilizers. Increasing agricultural production by harnessing the plant-associated microbiome is a tantalizing prospect. Prior to this, ways to change the composition and function of microbiomes need to be identified (“microbiomes engineering”). As mentioned, research efforts have been made on manipulating plant microbiomes by inducing the plant’s signaling defense pathways using exogenous phytohormone treatments. Nevertheless, the contradicting results obtained from different studies suggest that it is still challenging to manipulate the plant’s endosphere microbiome. Future studies need to be conducted to bridge the knowledge gaps of how microbial function of the endosphere is affected by plant immunity. A combination of multi ‘omics’ such as metagenomics, proteomics and metabolomics and the advancing computational data-mining approaches should be able to reveal a more comprehensive picture of the endosphere microbiome, therefore transforming the way we understand bacterial endophytes and their interactions with plant hosts. It must, however, be addressed that culture-dependent methods are still important because they provide the indispensable materials for identifying bacterial physiological characteristics and allow the predication of the metabolic potential and biogeochemical function of a lineage by using genomic surveys. Promising areas to develop are efforts to breed for endophyte-optimized crops, endophytic microbiomes engineering and a better understanding how key endophytes can be attracted, maintained and adapted to benefit plants at various growth stages. While substantial basic and applied work remains to be done, it is envisioned that in the not too distant future bacterial endophytes can be at least partial substitutes for chemical fertilizers and pesticides and their targeted applications on crops may push forward a paradigm shift in agriculture.

## Author Contributions

HL did the writing and drew the graphs. LC, MC, ES, PD, CP, and PS revised this manuscript.

## Conflict of Interest Statement

The authors declare that the research was conducted in the absence of any commercial or financial relationships that could be construed as a potential conflict of interest.
