# Supplementary material for: Inner Plant Values: Diversity, Colonization and Benefits from Endophytic Bacteria
Source: Front Microbiol. 2017 Dec 19;8:2552. doi: 10.3389/fmicb.2017.02552 (PMC5742157; doi:10.3389/fmicb.2017.02552)
Supplement: Supplementary file 1 [file Table_1.DOCX]

**Table S1** A table summarising proportions of bacterial phyla in plant endosphere based on 25 references using either culture- dependent (CD) or independent (CID) method.

**References** (1-9 are cited in the main manuscript of the review and 10-25 are listed below)

Chen, L., Luo, S., Chen, J., Wan, Y., Liu, C., Liu, Y., Pang, X., Lai, C., and Zeng, G. (2012). Diversity of endophytic bacterial populations associated with Cd-hyperaccumulator plant *Solanum nigrum* L. grown in mine tailings. *Appl Soil Ecol* 62**,** 24-30.

Costa, L.E.D.O., Queiroz, M.V.D., Borges, A.C., Moraes, C.a.D., and Araújo, E.F.D. (2012). Isolation and characterization of endophytic bacteria isolated from the leaves of the common bean (*Phaseolus vulgaris*). *Braz J Microbiol* 43**,** 1562-1575.

Ding, T., and Melcher, U. (2016). Influences of plant species, season and location on leaf endophytic bacterial communities of non-cultivated plants. *PloS one* 11**,** e0150895.

Jin, H., Yang, X.Y., Yan, Z.Q., Liu, Q., Li, X.Z., Chen, J.X., Zhang, D.H., Zeng, L.-M., and Qin, B. (2014). Characterization of rhizosphere and endophytic bacterial communities from leaves, stems and roots of medicinal *Stellera chamaejasme* L. *Syst Appl Microbiol* 37**,** 376-385.

Khan, Z., and Doty, S.L. (2009). Characterization of bacterial endophytes of sweet potato plants. *Plant Soil* 322**,** 197-207.

Liu, X.L., Liu, S.L., Liu, M., Kong, B.H., Liu, L., and Li, Y.H. (2014). A primary assessment of the endophytic bacterial community in a xerophilous moss (*Grimmia montana*) using molecular method and cultivated isolates. *Braz J Microbiol* 45**,** 165-173.

Luo, S.L., Chen, L., Chen, J.L., Xiao, X., Xu, T.Y., Wan, Y., Rao, C., Liu, C.B., Liu, Y.T., and Lai, C. (2011). Analysis and characterization of cultivable heavy metal-resistant bacterial endophytes isolated from Cd-hyperaccumulator *Solanum nigrum* L. and their potential use for phytoremediation. *Chemosphere* 85**,** 1130-1138.

Manter, D.K., Delgado, J.A., Holm, D.G., and Stong, R.A. (2010). Pyrosequencing reveals a highly diverse and cultivar-specific bacterial endophyte community in potato roots. *Microb Ecol* 60**,** 157-166.

Mitter, E.K., De Freitas, J.R., and Germida, J.J. (2017). Bacterial root microbiome of plants growing in oil sands reclamation covers. *Front Microbiol* 8.

Palaniappan, P., Chauhan, P.S., Saravanan, V.S., Anandham, R., and Sa, T. (2010). Isolation and characterization of plant growth promoting endophytic bacterial isolates from root nodule of *Lespedeza* sp. *Biol Fertil Soils* 46**,** 807-816.

Proença, D.N., Francisco, R., Kublik, S., Schöler, A., Vestergaard, G., Schloter, M., and Morais, P.V. (2017). The microbiome of endophytic, wood colonizing bacteria from pine trees as affected by pine wilt disease. *Sci Rep* 7.

Romero, F.M., Marina, M., and Pieckenstain, F.L. (2014). The communities of tomato (*Solanum lycopersicum* L.) leaf endophytic bacteria, analyzed by 16S-ribosomal RNA gene pyrosequencing. *FEMS Microbiol Lett* 351**,** 187-194.

Su, J., Ouyang, W., Hong, Y., Liao, D., Khan, S., and Li, H. (2016). Responses of endophytic and rhizospheric bacterial communities of salt marsh plant (Spartina alterniflora) to polycyclic aromatic hydrocarbons contamination. *J Soils Sediments* 16**,** 707-715.

Sun, L., Qiu, F., Zhang, X., Dai, X., Dong, X., and Song, W. (2008). Endophytic bacterial diversity in rice (*Oryza sativa* L.) roots estimated by 16S rDNA sequence analysis. *Microb Ecol* 55**,** 415-424.

Szymańska, S., Płociniczak, T., Piotrowska-Seget, Z., Złoch, M., Ruppel, S., and Hrynkiewicz, K. (2016). Metabolic potential and community structure of endophytic and rhizosphere bacteria associated with the roots of the halophyte *Aster tripolium* L. *Microbiol Res* 182**,** 68-79.

Thomas, P., and Sekhar, A.C. (2017). Cultivation versus molecular analysis of banana (*Musa* sp.) shoot-tip tissue reveals enormous diversity of normally uncultivable endophytic bacteria. *Microb Ecol* 73**,** 885-899.

Wemheuer, F., Kaiser, K., Karlovsky, P., Daniel, R., Vidal, S., and Wemheuer, B. (2017). Bacterial endophyte communities of three agricultural important grass species differ in their response towards management regimes. *Sci Rep* 7**,** 40914.

Xia, Y., Greissworth, E., Mucci, C., Williams, M.A., and De Bolt, S. (2013). Characterization of culturable bacterial endophytes of switchgrass (*Panicum virgatum* L.) and their capacity to influence plant growth. *Glob Change Biol Bioenergy* 5**,** 674-682.
